# Kikuchi‐Fujimoto disease: investigating comprehensive clinicopathological features and risk factors for recurrence

**DOI:** 10.1111/his.15427

**Published:** 2025-02-17

**Authors:** Midori Filiz Nishimura, Chikako Sakao, Yuka Kurokawa, Yoshito Nishimura, Asami Nishikori, Hidetaka Yamamoto, Yasuharu Sato

**Affiliations:** ^1^ Department of Molecular Hematopathology Okayama University Graduate School of Health Sciences Okayama Japan; ^2^ Department of General Medicine Okayama University Graduate School of Medicine, Dentistry, and Pharmaceutical Sciences Okayama Japan; ^3^ Division of Hematology and Oncology Mayo Clinic Rochester Minnesota USA; ^4^ Department of Pathology and Oncology, Graduate School of Medicine, Dentistry and Pharmaceutical Sciences Okayama University Okayama Japan

**Keywords:** histiocytic necrotizing lymphadenitis, histological subtypes, Kikuchi‐Fujimoto disease, necrotizing type, proliferating type, recurrent, xanthomatous type

## Abstract

**Aims:**

Kikuchi‐Fujimoto disease (KFD) is a rare disease that typically manifests with fever and cervical lymphadenopathy. Little is known about the risk factors associated with recurrence and their correlation with clinicopathologic features.

**Methods and Results:**

We analysed 112 patients with KFD, predominantly female (61/112, 54.5%), with an average age of 29.4 years. The incidence was higher in males up to the age of 20 and higher in females from their 30s onwards. Of the 70 patients with follow‐up data, 23% experienced recurrence. Recurrence was associated with lower C4 levels (*P* = 0.038) and higher antinuclear antibody (ANA) rates (*P* = 0.007) compared to transient disease. The mean duration of symptoms was 71.5 days. Lymph node histology in 98 cases (excluding 14 needle biopsy specimens) was classified into three patterns: proliferative (*n* = 75, 77%), necrotizing (*n* = 22, 22%), and xanthomatous (*n* = 1, 1%). The necrotizing pattern associated with significantly enlarged lymph nodes (*P* = 0.047) and a longer symptom duration (*P* = 0.009) than the proliferating pattern. The number of CD4‐positive lymphocytes was significantly lower in the necrotizing type than in the proliferative type (*P* < 0.001).

**Conclusion:**

These results indicated that low C4 levels and positive ANA were associated with KFD recurrence. Although the aetiology of KFD remains elusive, given that some cases develop autoimmune disease, the results suggest that patients with recurrent KFD represent an intermediate status between those with transient KFD and those with overt autoimmune disease. The comprehensive clinicopathological findings of this study may be useful for elucidating its pathogenesis and predicting the clinical course.

## Graphical abstract

Comprehensive clinicopathological analysis of 112 cases of Kikuchi‐Fujimoto disease (KFD) was conducted, and offered insights into recurrence risk factors and histopathological patterns. Recurrent KFD may fall between transient KFD and overt autoimmune disorders and lead to a better understanding of the aetiology of KFD.

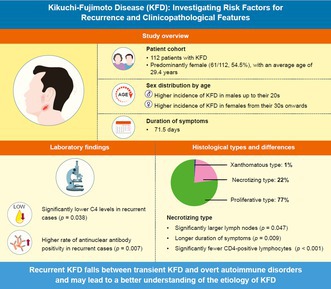

**Abbreviations**
ANA: antinuclear antibody
HPF: high‐power field
KFD: Kikuchi‐Fujimoto disease
LZM: Lysozyme
MPO: myeloperoxidase
PDC: Plasmacytoid dendritic cell
SLE: Systemic lupus erythematosus

## Introduction

Kikuchi‐Fujimoto disease (KFD) was first reported in Japan^1^, ^2^ and has a worldwide distribution; however, it is most frequently reported in Asian countries.^3^, ^4^ Histologically, it is characterized by paracortical lesions with varying degrees of necrosis, crescentic histiocytes containing nuclear debris, and CD8‐positive T cells. KFD has been included in the 5th Edition of the World Health Organization Classification of Hematolymphoid Tumours under “Tumour‐like lesions with T‐cell predominance” in the chapter “T‐cell and NK‐cell lymphoid proliferations and lymphomas”.^5^

KFD affects a wide range of age groups of both genders. Although KFD has previously been thought to primarily affect women under the age of 30, there have been reports of no apparent gender distribution,^6^ as well as reports of male predominance in paediatric patients.^7^, ^8^, ^9^ Additionally, different age groups and gender exhibit different clinical features.^8^

KFD usually has a transient clinical course, with symptoms spontaneously resolving within 1–4 months^10^, ^11^; however, some patients may experience recurrence. The reported frequency of recurrence varies (3%–10%),^8^, ^11^ with paediatric patients reported to have a higher recurrence rate than adults (up to 42.4%).^12^, ^13^, ^14^ Risk factors for KFD recurrence include a history of systemic disease, a higher absolute lymphocyte count in paediatric patients,^14^ and ANA positivity in adults^15^; however, no clinical factors have been established to predict KFD recurrence.

The lymph node histology of KFD varies and can be classified into three patterns: proliferative, necrotizing, and xanthomatous patterns.^16^ However, few studies have examined the differences in clinical findings between the respective histological patterns.

In this study we examined the clinical, laboratory, and histological findings in a large cohort of Japanese patients with KFD according to their age and gender. Factors associated with recurrence were also studied by comparing the clinical findings between the transient and recurrent groups.

## Materials and Methods

### Patients

**Figure 1:**
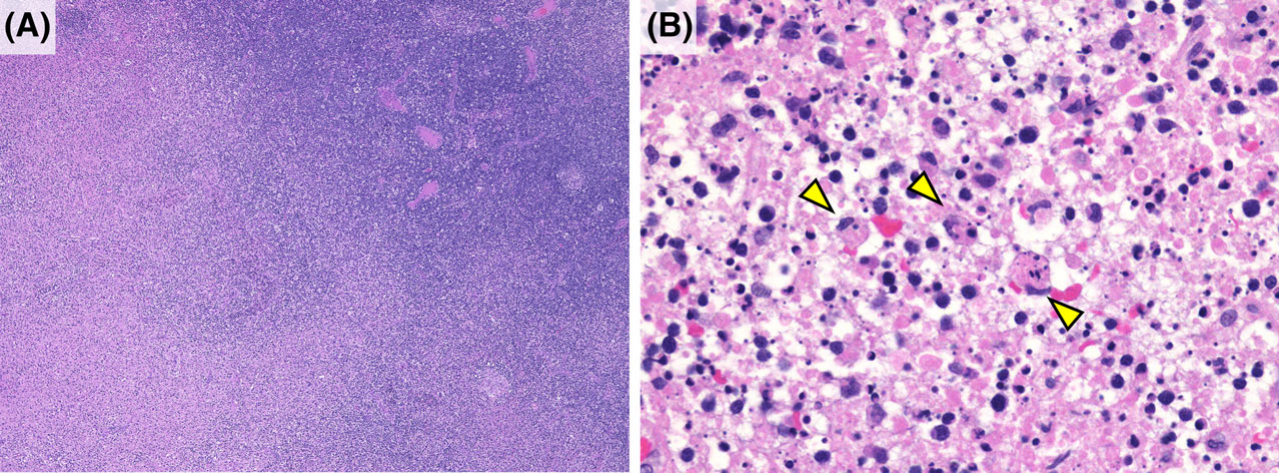
Histological features of KFD. (**A**) Eosinophilic lesions are observed in the paracortical area (lower left of the image) (H&E, ×40). (**B**) The lesion shows infiltration of large lymphocytes and crescentic histiocytes (arrowheads) against a background of eosinophilic debris and karyorrhectic nuclear fragments consistent with the necrotizing pattern of KFD^16^ (H&E, original magnifications ×400). [Color figure can be viewed at wileyonlinelibrary.com]

We retrieved 112 patient records with KFD (26 patients from Okayama University Hospital and 86 from city hospitals) diagnosed between January 2012 and August 2023 from the consultation file of the Okayama University Department of Pathology and Oncology and retrospectively analysed the data. All patients included in this study were Japanese. The diagnosis of KFD was confirmed based on the histological findings of the affected lymph nodes obtained by excisional or needle biopsy. The lymph nodes of all patients with KFD included in this study had multifocal lesions in the paracortical area, with histiocytes with crescentic nuclei engulfing karyorrhectic debris and without neutrophilic infiltration^16^, ^17^ (Figure 1). None of the cases met the classification criteria for systemic lupus erythematosus (SLE) or any other known autoimmune disease during the follow‐up period.

### Collection and analysis of clinical information

Clinical data collected from medical records are summarized in Table 1.

**Table 1:** Clinical data collected from medical records

Category	Details
Basic patient information	Age at diagnosis, gender
Localization of lymphadenopathy	Specific locations of lymphadenopathy, e.g., cervical, axillary, inguinal, etc.
Clinical symptoms	Fever, lymph node tenderness, skin rash
Laboratory findings	Complete Blood Count: white blood cells (WBC), platelets, haemoglobin, MCV, MCHC
	Biochemistry: soluble interleukin‐2 receptor (sIL‐2R), lactate dehydrogenase (LDH), C‐reactive protein (CRP), albumin, BUN, creatinine, complement levels (C3, C4, CH50), ferritin, immunoglobulin G (IgG)
	Autoantibodies: antinuclear antibody (ANA), anti‐sm antibody, anti‐dsDNA antibody, lupus anticoagulant, anticardiolipin antibody, direct coombs test
Clinical course	Recurrence, duration of symptoms, treatment details

In order to investigate the relationship between the histology and time course of the affected lymph nodes, symptom duration was defined as the period from the onset of lymphadenopathy to the date of symptom improvement based on medical records. Symptom improvement was considered when there was a noticeable improvement in all symptoms, including lymphadenopathy, fever, and skin rash. Lymphadenopathy was considered improved if there was a reduction in size, rather than complete resolution.

The recurrence of KFD was determined based on clinical evaluation and the symptom patterns documented in the medical records, with other potential causes for the symptoms ruled out. The recurrence was defined by fulfilling either of the following criteria:

1. Similar symptoms based on medical records: Recurrence of symptoms similar to those documented in previous episodes, as noted in the medical records, with at least a 2‐week interval between the current episode and the prior episode.
2. A new episode during the observation period: A recurrence occurred at least 2 weeks after the resolution of the previous episode in which a lymph node biopsy was performed.

### Evaluation of histological findings

**Figure 2:**
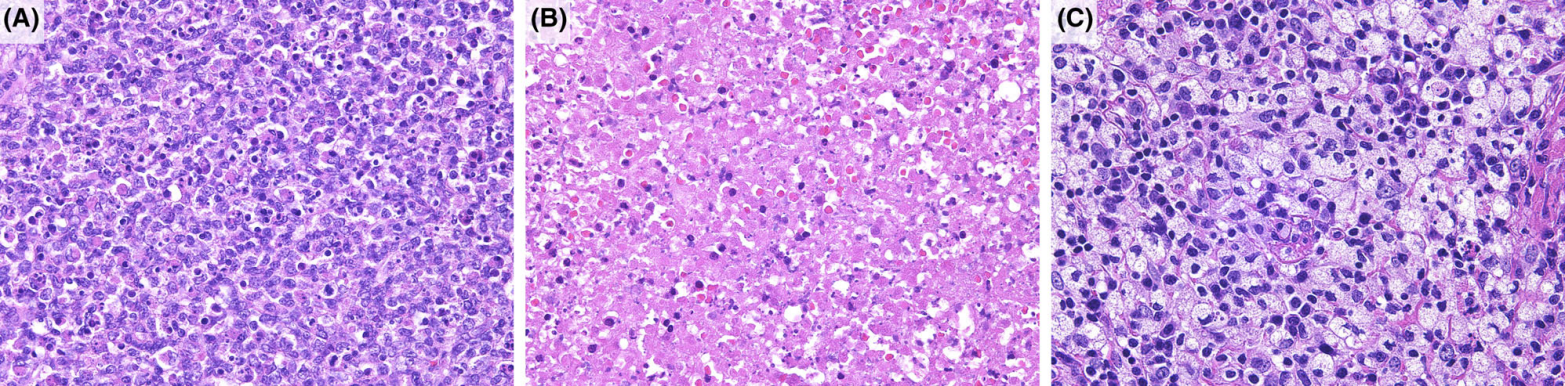
Representative images of the three histological patterns of KFD.^16^ (**A**) Proliferating pattern: the lesion comprises various infiltrates, including histiocytes, plasmacytoid dendritic cells, lymphocytes, and karyorrhectic debris (H&E, ×400). (**B**) Necrotizing pattern: the lesion includes areas of coagulation necrosis (H&E, ×400). (**C**) Xanthomatous pattern: foamy histiocytes predominate in the lesions (H&E, ×400). [Color figure can be viewed at wileyonlinelibrary.com]

The histological evaluation was performed by two independent hematopathologists (M.F.N. and Y.S.). Based on the previous report,^16^ lymph node histological patterns were classified as proliferative, necrotizing, and xanthomatous. Each histological pattern was classified according to the following characteristics: (1) proliferative, if the lesion was composed of various histiocytes, plasmacytoid dendritic cells, and lymphocytes in the background of karyorrhectic nuclear fragments and eosinophilic debris; (2) necrotizing, if the lesion was accompanied by coagulative necrosis to any degree within the cellular aggregates; and (3) xanthomatous if foamy histiocytes predominated in the lesion, regardless of the presence or absence of necrosis. The size, clinical course, and laboratory findings of the biopsied lymph nodes were compared based on the histopathological patterns. Figure 2 shows the representative histological images of the three patterns. Histological examination of haematoxylin and eosin (H&E)‐stained lymph node sections was performed to classify histological patterns. The analysis of the histological pattern and evaluation of immunostaining were performed on 98 cases, excluding the 14 needle biopsy specimens, due to the inability to observe the entire lesion.

Immunohistochemistry was performed using an automated Bond III instrument (Leica Biosystems, Wetzlar, Germany) with the following primary antibodies: myeloperoxidase (MPO) (polyclonal, 1:2000; Dako, Agilent, Santa Clara, CA, USA), lysozyme (LZM) (polyclonal, 1:2000; Dako), CD4 (IFI6, 1:50; Nichirei, Japan), CD8 (C8/144B, 1:200; Nichirei, Japan), and CD123 (BR4MS, 1:100; Leica Biosystems, Germany). CD4‐positive and CD8‐positive cells were evaluated in the affected area of KFD and represented the average of three high‐power field (HPF)s, while CD123‐positive cells were evaluated in the areas of highest density among the affected lymph nodes and represented the average of three HPFs. The CD4/CD8‐positive cell ratio was calculated by counting the number of positive cells in the same field at three different locations and averaged only in cases where both CD4 and CD8 immunostained specimens were available.

### Statistical analysis

The data were analysed using the R Studio software (R v. 4.3.0, Vienna Austria). Continuous variables were compared using the Wilcoxon rank‐sum test, and categorical variables were analysed using Fisher's exact test. A *P*‐value <0.05 was considered statistically significant.

### Ethics statement

This study was approved by the Institutional Review Board of Okayama University (protocol number 2310‐029; approved on September 8, 2023). All procedures were conducted in accordance with the tenets of the Declaration of Helsinki. Because this was a retrospective analysis of existing pathology samples, an opt‐out method of informed consent was used: patients were provided with information about the study and given the opportunity to decline participation.

## Results

### Clinical findings

Table 2 summarizes the demographics and clinical findings of the 112 patients with KFD. The mean age of the patients at diagnosis was 29.1 years. No gender differences were found among the patients (male‐to‐female ratio, 51:61).

**Table 2:** Demographic and clinical features of patients with KFD

Characteristics	*n* = 112
Age (mean ± SD)	29.1 ± 12.5
Sex (M:F)	51:61
Lymphadenopathy *n* (%)	
Unifocal (one LN station)	59 (53)
Multifocal (two or more LN stations)	53 (47)
Localization	
Cervical	99 (88)
Submandibular	10 (9)
Clavicular	28 (25)
Axillary	23 (21)
Paratracheal/hilar	5 (4)
Abdominal	7 (6)
Paraaortic	5 (4)
Inguinal	13 (12)
Other	12 (11)
Symptoms *n* (%)	
Fever	68 (61)
Tenderness of LNs	43 (38)
Skin rash	12 (11)

LN, lymph node; SD, standard deviation.

**Figure 3:**
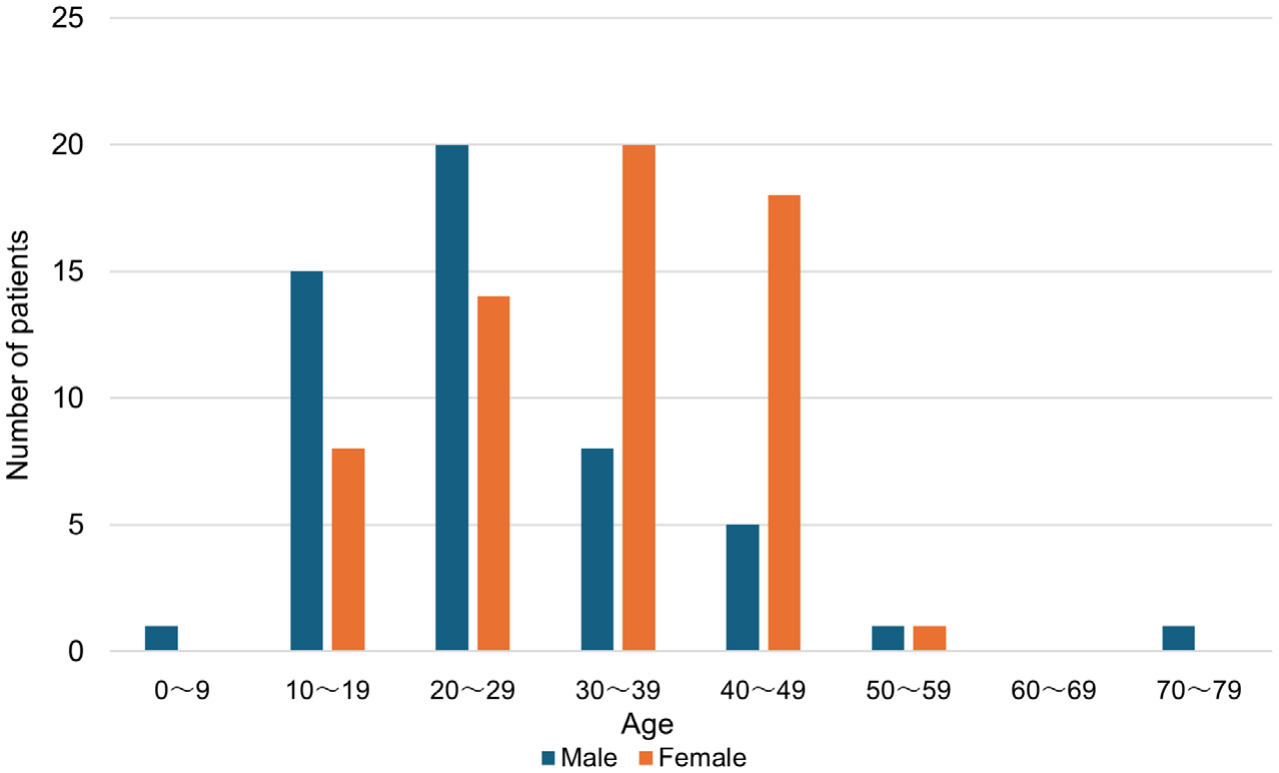
Distribution of age at diagnosis of KFD shown by gender. Overall, the incidence peaked among individuals in their 20s. A male predominance was observed up to their 20s, while a female predominance was noted from their 30s onwards. [Color figure can be viewed at wileyonlinelibrary.com]

The age distribution at diagnosis (Figure 3) showed a male predominance up to their 20s and a female predominance in those in their 30s and older.

Lymphadenopathy was unifocal (restricted to one lymph node station) in 59 patients (53%), and multifocal (≥ two lymph node stations) in 53 patients (47%), with cervical lymphadenopathy being the most common (99 patients, 88%). Fever, lymph node tenderness, and rashes were observed in 68 (61%), 43 (38%), and 12 (12%) patients, respectively.

### Laboratory findings

**Figure 4:**
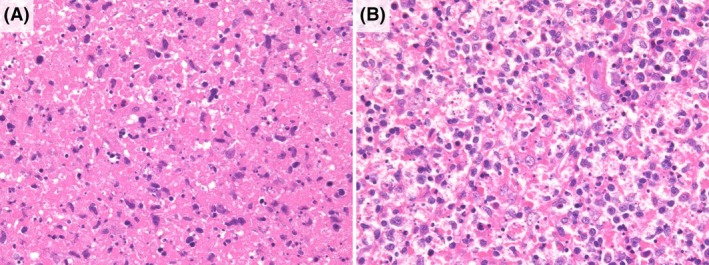
Pathological findings in patients with positive autoantibodies. (**A**) Histological findings in anti‐Sm antibody‐positive patients. The histology showed a necrotizing pattern of KFD (H&E, ×400). (**B**) Histological findings in anti‐dsDNA antibody‐positive patient. The histology shows a proliferating pattern of KFD (H&E, ×400). [Color figure can be viewed at wileyonlinelibrary.com]

Table 3 shows the laboratory findings of the patients with KFD. Laboratory tests showed leukopenia in 63/93 (68%), elevated CRP (>2.0 mg/dL) in 12/81 (15%), elevated LDH (>500 IU/L) in 17/91 (19%), and elevated sIL‐2R (>800 U/mL) in 22/77 (29%) patients. Regarding the positive rate of autoantibodies, 8/46 (17%) of the KFD patients had positive ANA, defined as a titre ≥1:40 by the fluorescent antibody technique. However, the absolute value of the titre was only slightly elevated at 1:50 ± 17.3 (mean ± SD), Anti‐Sm antibody, anti‐dsDNA antibody, and anticardiolipin antibody were also positive in one case each. The clinical and histological findings of patients who tested positive for anti‐Sm or anti‐dsDNA antibodies are shown in Table 4 and Figure 4. MPO‐ and LZM‐positive crescentic histiocytes were observed in both anti‐Sm antibody‐positive and anti‐dsDNA antibody‐positive patients. In addition, the histological findings reported in SLE, such as the presence of haematoxylin bodies, neutrophilic infiltration, inflammatory cell infiltration of the vessel wall, and epithelial granulomas, were not observed, showing no difference in histology between autoantibody‐negative KFD patients.

**Table 3:** Laboratory findings of KFD

Findings	All KFD patients	Patients available for follow‐up [*n* = 70]		*P*
		Transient [*n* = 54]	Recurrent [*n* = 16]	Transient vs. recurrent
*Normal range*				
WBC (×10^3^/μL) *3.3–8.6*	3.7 ± 1.6 [*n* = 93]	3.5 ± 1.6	3.7 ± 1.6	0.898
Leukopenia, *n* (%)	63 (68)	39 (72)	11 (69)	0.763
Plt (×10^3^/μL) *158–348*	203.4 ± 68.2 [*n* = 85]	203 ± 66.7	194.4 ± 64.1	0.706
Hb (g/dL)	13.7 ± 3.4 [*n* = 85]	14.2 ± 3.9	12.7 ± 1.6	―
Male *13.7–16.8*	15.1 ± 4.3 [*n* = 39]	15.5 ± 5.0	13.8 ± 0.75	0.203
Female *11.6–14.8*	12.5 ± 1.5 [*n* = 46]	12.9 ± 1.2	12.2 ± 1.7	0.212
MCV *83.6–98.2*	86.2 ± 12.4 [*n* = 56]	86.6 ± 4.6	85.2 ± 6.3	0.67
MCHC *31.7–35.3*	33.6 ± 4.7 [*n* = 55]	33.6 ± 1.2	33.7 ± 1.8	0.379
CRP (mg/dL) *<0.30*	1.3 ± 2.8 [*n* = 81]	0.86 ± 1.6 [*n* = 53]	0.71 ± 0.86 [*n* = 15]	0.647
CRP >2.0 mg/dL *n* (%)	12 (15)	7 (13)	1 (7)	0.670
LDH (IU/L) *120–240*	387.5 ± 434.9 [*n* = 91]	327.6 ± 197.3 [*n* = 52]	274.8 ± 158.8	0.313
LDH >500 IU/L *n* (%)	17 (19)	7 (13)	2 (13)	1
sIL‐2R (U/mL) *157–475*	818.2 ± 985.7 [*n* = 77]	672.3 ± 374.0 [*n* = 41]	528.3 ± 269.5 [*n* = 12]	0.2
sIL‐2R >800 U/mL *n* (%)	22 (29)	12 (29)	2 (17)	0.48
Alb (g/dL) *3.9–4.9*	4.1 ± 0.57 [*n* = 70]	4.1 ± 0.5 [*n* = 47]	4.0 ± 0.48 [*n* = 14]	0.232
BUN (mg/dL) *8.1–22.0*	11.5 ± 3.2 [*n* = 76]	11.6 ± 3.3 [*n* = 52]	11.2 ± 2.9	0.816
Cre (mg/dL) *<1.0*	0.69 ± 0.15 [*n* = 77]	0.70 ± 0.15 [*n* = 52]	0.65 ± 0.17	0.082
C3 (mg/dL) *65–135*	117.4 ± 22.9 [*n* = 27]	119.0 ± 23.2 [*n* = 16]	107.7 ± 17.7 [*n* = 7]	0.315
C4 (mg/dL) *13–35*	32.5 ± 12.0 [*n* = 27]	37.2 ± 10.0 [*n* = 16]	24.5 ± 12.1 [*n* = 7]	**0.038**
CH50 (mg/dL) *30–50*	55.9 ± 14.0 [*n* = 28]	59.3 ± 14.4 [*n* = 19]	49.0 ± 11.4 [*n* = 6]	0.180
Ferritin (μg/mL)	310 ± 418 [*n* = 41]	289 ± 319 [*n* = 29]	359 ± 588 [*n* = 12]	―
Male *39.9–465*	565 ± 533 [*n* = 21]	453 ± 340 [*n* = 16]	712 ± 770 [*n* = 5]	0.910
Female *6.2–138*	83.4 ± 82.3 [*n* = 20]	70.0 ± 38.2 [*n* = 13]	107 ± 125 [*n* = 7]	0.901
IgG (mg/dL) *870–1818*	1377 ± 278 [*n* = 33]	1401 ± 266 [*n* = 20]	1296.5 ± 353.7 [*n* = 7]	0.818
ANA				
Positive *n*/*N* (%)	8/46 (17)	2/30 (7)	4/7 (57)	**0.007**
Titre (FAT)	50 ± 17.3	40	53.3 ± 18.9	―
Homogeneous *n*(%)	4 (50)	0 (0)	4 (100)	―
Speckled *n* (%)	6 (75)	1 (50)	3 (75)	―
Nuclear *n* (%)	2 (25)	0 (0)	0 (0)	―
Anti‐Sm antibody positive *n*/*N* (%)	1/13 (8)	0/6 (0)	0/5 (0)	―
Anti‐dsDNA antibody positive *n*/*N* (%)	1/18 (6)	0/8 (0)	0/6 (0)	―
LA positive *n*/*N* (%)	0/1 (0)	0/0 (0)	0/0 (0)	―
aCL positive *n*/*N* (%)	1/4 (25)	0/2 (0)	1/2 (50)	―
DAT positive *n*/*N* (%)	0/3 (0)	0/2 (0)	0/1 (0)	―

Data are shown as mean ± SD or percentage. *N* indicates the total number of patients who underwent the test. Leukopenia was defined by deviation from the reference values at each institution.

aCL, anticardiolipin antibody; ANA, antinuclear antibodies; CRP, C‐reactive protein; DAT, direct antiglobulin test; dsDNA, double‐stranded DNA; FAT, Fluorescent antibody technique; Hb, haemoglobin; LA, Lupus Anticoagulant; LDH, Lactate dehydrogenase; MCHC, mean corpuscular haemoglobin concentration; MCV, mean corpuscular volume; Plt, platelet; sIL‐2R, soluble interleukin‐2 receptor; WBC, white blood cells.

Bold values indicate statistically significant results (*P* < 0.05)

**Table 4:** Clinical findings in patients with positive anti‐Sm or anti‐dsDNA antibodies

Findings	(A)	(B)
	Anti‐Sm antibody‐positive patient	Anti‐dsDNA antibody‐positive patient
Age	32	6
Gender	F	M
Site of lymphadenopathy	Cervical, Axillary	Cervical
Symptoms	Fever, pleural effusion	Fever
Clinical course of KFD	Recurrent	RPecurrent
Laboratory data *Normal range*		
WBC (×10^3^/μL) *3.3–8.6*	3600	5000
Hb (g/dL) Male *13.7–16.8* Female *11.6–14.8*	13	11.5
Plt (×10^3^/μL) *158–348*	275	311
CRP (mg/dL) <0.30	14.3	NA
C3 (mg/dL) *65–135*	NA	88.2
C4 (mg/dL) *13–35*	NA	13.9
ANA	Positive (Homogeneous, Speckled)	NA
ANA titre (FAT)	40	NA
Anti‐Sm antibody	+	−
Anti‐dsDNA antibody	−	+

(A) Findings in an Anti‐Sm antibody‐positive patient. Pleural effusion and ANA positivity were observed; however, the patient did not meet any classification criteria for autoimmune diseases. (B) Findings in an anti‐dsDNA antibody‐positive patient. Laboratory findings showed decreased C4 levels, but did not meet any classification criteria for autoimmune diseases.

ANA, antinuclear antibodies; CRP, C‐reactive protein; FAT, fluorescent antibody technique; Hb, haemoglobin; Plt, platelet; WBC, white blood cells.

The patients were divided into transient (*n* = 54) and recurrent (*n* = 16) groups, and their laboratory findings were compared. The recurrent group showed significantly lower C4 levels (*P* = 0.038) and higher ANA positivity (*P* = 0.007). Although the haemoglobin level was significantly lower in the recurrent group, no significant difference was found when stratified by gender. No significant differences were observed between the transient and recurrent groups in other laboratory parameters.

### Clinical course

The clinical course and treatment of 70 patients with KFD were examined according to gender (Table 5). The follow‐up period (mean ± SD) was 172 ± 415.9 days. The overall recurrence rate was 23%, slightly higher in females (30%) than in males (15%); however, this difference was not statistically significant (*P* = 0.167). Of the 16 patients who presented with recurrence, five were male (mean age 18.8 years; range 10–27 years) and 11 were female (mean age 32.9 years; range 20–49 years). Forty‐four percent of patients with recurrence had two or more recurrences. The mean symptom duration was 71.5 days, with no significant gender differences. The mean duration from the previous episode to recurrence was 1035 days, with no significant gender differences. Nonsteroidal antiinflammatory drugs (NSAIDs) were the most commonly used treatment (43%), followed by antibiotics (33%), oral corticosteroids (27%), and acetaminophen (16%). Spontaneous remission with no treatment was observed in 26% of patients.

**Table 5:** Clinical course and medication details of KFD

Patients available for follow‐up [*n* = 70]		Male [*n* = 33]	Female [*n* = 37]	*P*
				*male vs. female*
Clinical course	Number of recurrent cases *n*/*N* (%)	Total		
		16/70 (23)		
		Male	Female	
		5/33 (15)	11/37 (30)	0.167
	Recurrence two or more times *n*/*N* (%)	Total		
		7/16 (44)		
		Male	Female	
		3/5 (60)	4/11 (36)	0.600
	Duration of symptoms (days) (mean ± SD)	Total		
		71.5 ± 73.9		
		Male	Female	
		83.3 ± 85.9	64.2 ± 48.3	0.891
	Duration from previous episode to recurrence (days) (mean ± SD)	Total		
		1035 ± 1094		
		Male	Female	
		928 ± 623	1089 ± 1261	0.806
Medication *n* (%)	NSAIDs	30 (43)		―
	Antibiotics	23 (33)		―
	Oral corticosteroid	19 (27)		―
	Acetaminophen	11 (16)		―
	No medication	18 (26)		―

*N* indicates the number of the population.

NSAIDS, nonsteroidal antiinflammatory drugs; SD, standard deviation.

### Comparison of clinical characteristics by histopathological pattern

In all 112 cases, including needle biopsy specimens, both MPO‐positive and LZM‐positive histiocytic infiltrate was observed, which is typical of the histology of KFD. For the 98 excised specimens, excluding 16 needle biopsied specimens, the histological patterns were classified according to a previous report^16^ (Figure 2). Table 6 presents a comparison of the size of the excised lymph nodes, clinical course, and laboratory findings according to histological pattern. The histopathological patterns were proliferative (75/98, 77%), necrotizing (22/98, 22%), and xanthomatous (1/98, 1%). The necrotizing type had significantly larger lymph node size (*P* = 0.047) and longer symptom duration (*P* = 0.004) than the proliferative type. No significant differences were observed in recurrence rate, days from symptom onset to biopsy, days from biopsy to improvement, or laboratory findings between the histopathological patterns.

**Table 6:** Comparison of findings by histological patterns of KFD

Findings	Histological patterns of KFD [*n* = 98]			*P*
	Proliferative *n* = 75 (77%)	Necrotizing *n* = 22 (22%)	Xanthomatous *n* = 1 (1%)	Proliferative vs necrotizing
LN size				
Short diameter of excited LNs (mm) (mean ± SD)	7.8 ± 4.2	10.5 ± 5.1	8	**0.047**
Clinical findings				
Number of recurrent cases *n*(%)	11 (15)	1 (4)	0 (0)	0.2869
Duration of symptoms (days)	60.9 ± 70.5 [*n* = 42]	118.5 ± 72.8 [*n* = 10]	45 [*n* = 1]	**0.009**
Duration from the onset of symptoms to the date of LN biopsy (days)	51.8 ± 68.4 [*n* = 48]	60.9 ± 46.8 [*n* = 15]	30 [*n* = 1]	0.175
Duration from LN biopsy to improvement of symptoms (days)	26.2 ± 24.2 [*n* = 38]	62.6 ± 53.1 [*n* = 9]	14 [*n* = 1]	0.131
Laboratory findings (mean ± SD)				
LDH (IU/L)	353.6 ± 413.3	584.2 ± 569.9	268 (100)	0.354
sIL‐2R (U/mL)	751.9 ± 991.5	1211.4 ± 1142.7	500 (0)	0.246
CRP (mg/dL)	0.85 ± 1.5	3.4 ± 5.2	0.35 (100)	0.505
ANA positive, *n*/*N*(%)	6/31 (19)	1/10 (10)	NA	0.659

The histological pattern of KFD was evaluated in 98 cases, excluding 14 needle biopsies from 112 cases.

ANA, antinuclear antibodies; CRP, C‐reactive protein; LDH, lactate dehydrogenase; LN, lymph node; NA, not available; SD, standard deviation; sIL‐2R, soluble interleukin‐2 receptor.

Bold values indicate statistically significant results (*P* < 0.05)

We further examined the differences in the numbers of CD4‐ or CD8‐positive lymphocytes and CD123‐positive cells, a marker for plasmacytoid dendritic cells (PDCs), between the proliferative and necrotizing types. We counted the number of positive cells in patients for whom immunostaining was available (22 cases for CD4, 35 cases for CD8, and 34 cases for CD123 in the proliferative type; and 8 cases for CD4, 10 cases for CD8, and 10 cases for CD123 in the necrotizing type).

**Figure 5:**
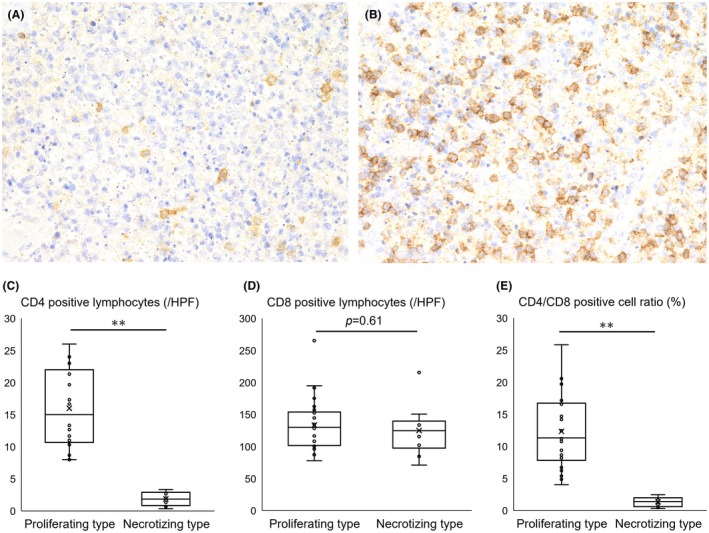
CD4‐positive cells and CD8‐positive cells in KFD lesions. (**A**) CD4‐positive lymphocytes are scattered sparsely (CD4 staining, ×400), and (**B**) CD8‐positive lymphocytes are more numerous than CD4‐positive cells (CD8 staining, ×400). (**C**) The number of CD4‐positive cells was significantly lower in the necrotizing pattern, and (**D**) there was no difference in the number of CD8‐positive cells between the two groups. (**E**) CD4/CD8‐positive cell ratio was significantly lower in the necrotizing pattern than in the proliferating pattern. Data are shown as boxplots, with whiskers representing the minimum to maximum. Differences between the two groups were evaluated using the Mann–Whitney *U* test (**P < 0.001). HPF, high‐power field. [Color figure can be viewed at wileyonlinelibrary.com]

In the areas of KFD lesions, CD8‐positive cells were predominant over CD4‐positive cells in both the proliferating and necrotizing types (Figure 5A,B). The number of CD4‐positive cells was significantly lower in the necrotizing type, while the number of CD8‐positive cells was not different between the two types; therefore, the CD4/CD8‐positive cell ratio was also significantly lower in the necrotizing type (Figure 5C,D).

**Figure 6:**
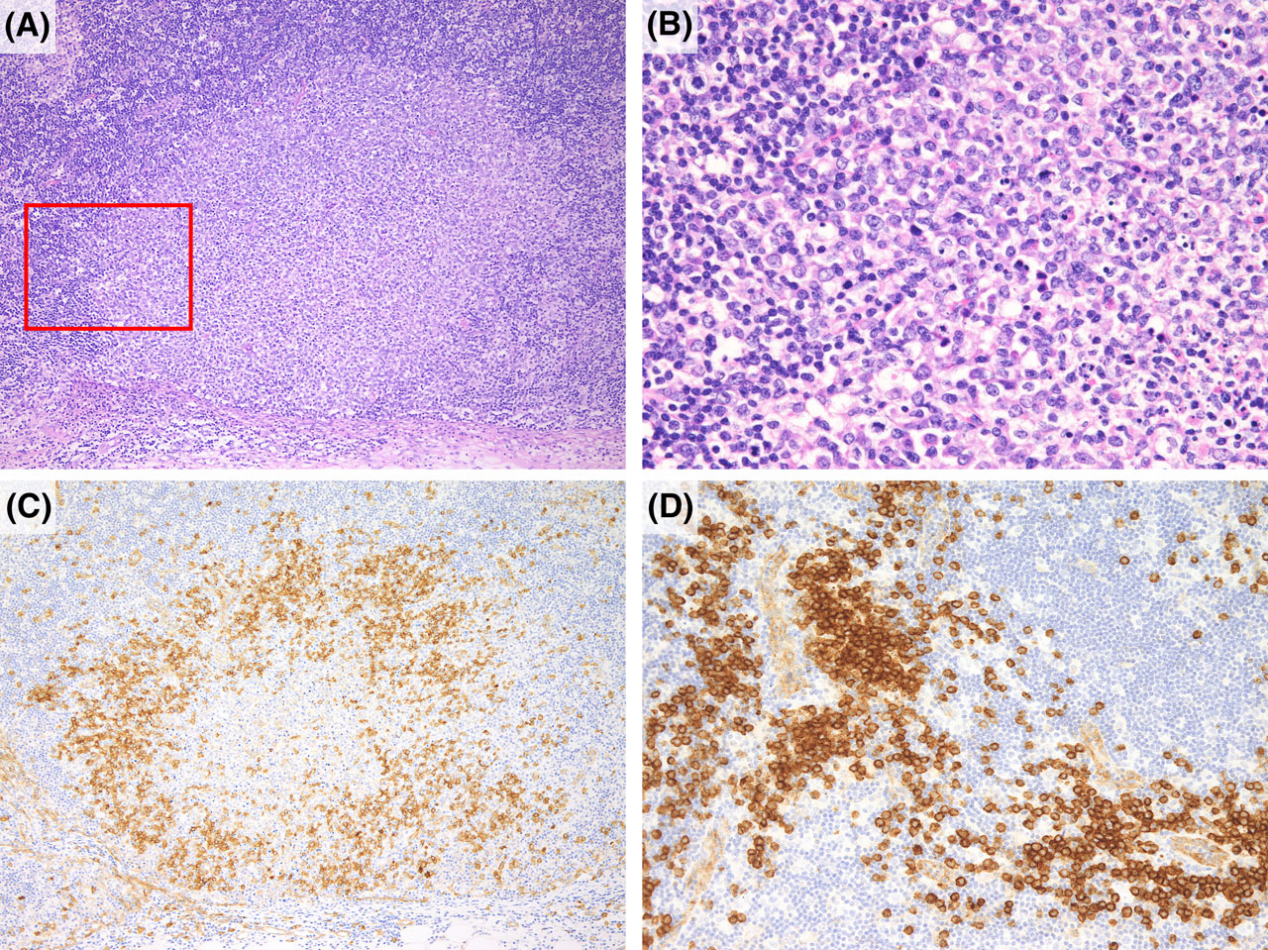
Distribution of plasmacytoid dendritic cells (PDC)s. (**A**) Patchy lesion in a KFD‐affected lymph node (centre) (H&E, ×100). (**B**) Magnified view of the red square in (**A**). PDCs with round nuclei are distributed along the margins of the lesion (H&E, ×400). (**C**) CD123 staining of the same microscopic field as (**A**). The distribution of CD123‐positive PDCs at the margins of the KFD lesion is highlighted (H&E, ×100). (**D**) CD123 staining of a lymph node from another patient with KFD. CD123‐positive cells are distributed around the high‐endothelial venules (H&E, ×200). [Color figure can be viewed at wileyonlinelibrary.com]

**Figure 7:**
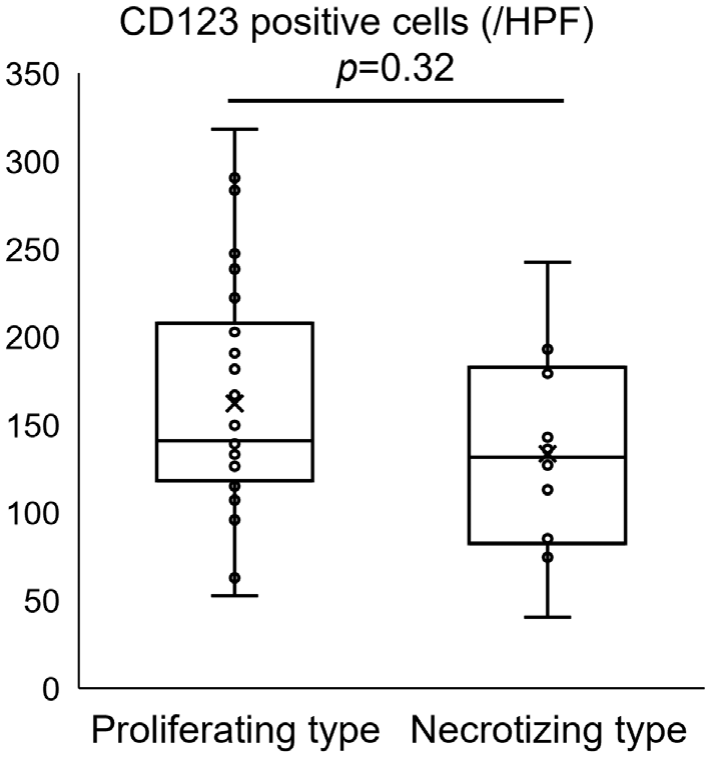
Comparison of CD123‐positive plasmacytoid dendritic cell (PDC) counts among histological patterns in KFD. The number of CD123‐positive cells did not differ significantly between the proliferating and necrotizing patterns. Data are shown as boxplots, with whiskers representing the minimum to maximum. Differences between the two groups were evaluated using the Mann–Whitney U test (**P < 0.001). HPF, high‐power field.

CD123‐positive PDCs were distributed in clusters around necrotic areas and/or high endothelial venules in both types (Figure 6). There was no difference in the number of CD123‐positive cells between the two histological types (Figure 7).

## Discussion

KFD has been reported mainly in Asian countries^3^, ^4^, ^10^ and is known to predominantly affect women in their 30s and 40s.^18^ However, the male–female predominance in incidence has also been reported to vary with age, with some reports indicating a male predominance in paediatric patients.^8^, ^19^, ^20^, ^21^ The present results, combined with the existing literature, suggest that KFD may be more likely to occur in males up to the age of 20 years and in females from the age of 30 years onward. The reason for the reversal of gender distribution by age is unclear; differences in immune responses at different ages and genders may be involved, warranting further investigation. Differences in disease epidemiology based on age group may help clinicians identify KFD cases in paediatric patients.

In the present study the overall recurrence rate was 23%. Previous reports have indicated that the recurrence rate of KFD is ~3%–10%,^3^, ^8^, ^22^, ^23^ but in paediatric KFD, the recurrence rate has been reported as high as 42%.^14^ In our cohort, the 70 patients with available clinical follow‐up data included 13 (18.6%) paediatric patients aged <18 years. There was no statistically significant difference between recurrence rates in the paediatric and older age groups; however, further validation is warranted. It should be noted that the definition of recurrence differs in the literature, which may have influenced the differences in the reported recurrence rates. A standardized definition of recurrence must be established to enable comparison across studies.

In a multivariate analysis, Yoo *et al*. suggested that a history of systemic illnesses and higher absolute lymphocyte count were risk factors for recurrent KFD.^14^ However, laboratory indicators for predicting KFD recurrence have not yet been established.

Our results suggest that decreased C4 levels and ANA positivity may be risk indicators of KFD recurrence. C4 is one of the early components of the classical complement pathway and can be reduced either by consumption during activation of the classical complement pathway by immune complexes or by decreased production of the C4 protein. Recent studies have also suggested an association between KFD recurrence and autoimmune disease,^24^ with 21%–36% of the recurrent group developing autoimmune disease.^25^, ^26^ Another report suggested that the abundance of nuclear debris in KFD‐affected lymph nodes increases the likelihood of autoantigen release, leading to the production of autoantibodies and the onset of autoimmune diseases.^19^ In KFD, particularly in recurrent cases, immune complexes may be produced and contribute to pathogenesis. The absence of a significant reduction in C3 and CH50 may further suggest that the number of immune complexes is lower than in overt autoimmune diseases such as SLE.

Interestingly, lymphadenopathy in patients with SLE has been reported to exhibit histological findings quite similar to those of KFD.^27^, ^28^ Further, transcriptomic analysis indicates that both SLE and KFD involve dysregulation of the type I interferon pathway,^29^ suggesting that at least part of their pathogenesis is shared. Recurrent KFD with positive ANA and decreased C4 levels may represent an intermediate pathophysiology between transient KFD and overt SLE, making it a compelling subject for future molecular analysis.

Regarding the correlations between clinical and pathological features, the necrotizing type had significantly larger lymph nodes and longer symptom duration than the proliferative type. Although no significant differences were observed in laboratory findings, there was a tendency for higher LDH, CRP, and sIL‐2R levels in the necrotizing type than in the other histopathological patterns. This may be due to the extensive inflammatory responses associated with necrosis, which is related to the size of the lymph nodes and symptom duration. In paediatric KFD, a larger lymph node diameter has been reported to be significantly associated with a longer fever duration,^12^ which may be related to the tendency for larger lymph node size and longer symptom duration in the necrotizing type in this study.

Although there was no significant difference in the number of CD8‐positive T cells and CD123‐positive PDCs in the necrotizing lesions compared to the proliferating lesions, the number of CD4‐positive cells was significantly lower in the necrotizing lesions than in the proliferating lesions. KFD is characterized by the apoptosis of CD4^+^ T cells,^30^ and it has been reported that the number of CD4+ cells in affected lymph nodes decreases 2–4 weeks after onset and then gradually increases.^31^ The results support the notion that the progression of KFD leads to CD4^+^ T cell apoptosis.

Compared to the proliferative pattern, the necrotizing pattern exhibited a trend toward a longer duration from the onset of lymphadenopathy to lymph node biopsy, although not statistically significant, and a significant decrease in CD4‐positive cell count in the lesions. These histological patterns may reflect different stages of KFD progression, with the necrotizing pattern indicating a more advanced stage than the proliferative pattern.

Our study has several limitations. KFD patients who underwent biopsy may not be typical; KFD can be clinically diagnosed without biopsy in general, and patients who underwent biopsy (especially those who visited a university hospital) may have had a longer time to remission or had repeated recurrences, which may have been influenced by sampling bias.

In conclusion, we summarized comprehensive clinicopathological data from a large cohort of patients with KFD. Our results revealed that ANA positivity and decreased C4 levels may be risk factors for KFD recurrence. Additionally, we identified the association between histological variations and clinical findings. Further molecular investigations are warranted to gain a deeper understanding of the pathophysiology of KFD, particularly in relation to autoimmune diseases.

## Author contributions

CS, YK, and MFN analysed the data. CS, YK, MFN, and YN performed the literature review. MFN, AN, and YS performed pathological evaluations. MFN and CS wrote the article. YS and HY supervised the study and edited the article. All the authors have reviewed and approved the final article.

## Funding information

This work was supported by the Okayama Medical Foundation and Teraoka Scholarship Foundation.

## Conflict of interest

The authors declare no conflict of interest.

## Data Availability

The data that support the findings of this study are available from the corresponding author upon reasonable request.
